# Stereoscopic Offset Makes Objects Easier to Recognize

**DOI:** 10.1371/journal.pone.0129101

**Published:** 2015-06-16

**Authors:** Baptiste Caziot, Benjamin T. Backus

**Affiliations:** 1 Graduate Center for Vision Research, SUNY College of Optometry, 33 W. 42^nd^ St., New York, New York, 10036, United States of America; 2 SUNY Eye Institute, 33 W. 42^nd^ St., New York, New York, 10036, United States of America; Centre de Neuroscience Cognitive, FRANCE

## Abstract

Binocular vision is obviously useful for depth perception, but it might also enhance other components of visual processing, such as image segmentation. We used naturalistic images to determine whether giving an object a stereoscopic offset of 15-120 arcmin of crossed disparity relative to its background would make the object easier to recognize in briefly presented (33-133 ms), temporally masked displays. Disparity had a beneficial effect across a wide range of disparities and display durations. Most of this benefit occurred whether or not the stereoscopic contour agreed with the object’s luminance contour. We attribute this benefit to an orienting of spatial attention that selected the object and its local background for enhanced 2D pattern processing. At longer display durations, contour agreement provided an additional benefit, and a separate experiment using random-dot stimuli confirmed that stereoscopic contours plausibly contributed to recognition at the longer display durations in our experiment. We conclude that in real-world situations binocular vision confers an advantage not only for depth perception, but also for recognizing objects from their luminance patterns and bounding contours.

## Introduction

When seen binocularly, a point in the world may project to different locations on the two retinae. This binocular disparity provides a cue for depth perception [1]. However, any system that maximizes performance should make use of the available information, so if disparities are useful for other visual functions besides seeing in depth, they ought to be used for those functions. Any such use of disparity would be limited if disparity extraction were slower than the time course for extracting other signals such as luminance, orientation, or color, as is often assumed. However we recently showed that stereoscopic information starts to become available for perceptual decisions as early as luminance information, suggesting that disparity might play a more important role in intermediate visual processes than previously assumed [2].

It is clear that binocular vision is useful to estimate depth intervals, but it could be more generally useful. Does it contribute to object recognition? Béla Julesz speculated that this might be the case, after showing that disparity alone is sufficient to break an object’s camouflage and reveal its contour in random-dot stimuli [3,4]. Testing whether disparity might regularly contribute to object recognition under natural viewing conditions would best be accomplished using briefly presented naturalistic objects and scenes, which is the purpose of this study.

Previous studies have looked at the contribution of stereoscopic shape information to object recognition, that is, 3D information specified by variation in disparity across the object itself. Some studies found a contribution of stereopsis to static object recognition [5,6], while others did not [7–11]. We addressed a different question: whether placing an object stereoscopically in front of its background makes it easier to recognize. The objects in our experiments were stereoscopically flat, so disparity modulation across the object could not provide a classical 3D shape cue. In order to control recognition performance we presented the objects at randomly chosen locations in perifoveal vision (12 degrees eccentricity), and at this visual eccentricity, within-object disparity modulation cannot be measured by the visual system with precision (see Discussion).

Stereopsis could facilitate object recognition in at least two ways not related to the perception of distance *per se*. First, stereopsis supports pop-out in visual search tasks [12], which is usually interpreted as an orienting of attention to the location of the target [13,14]. In real-world situations, it could be important to quickly determine the visual field location and/or disparity of objects that are closer than the background, in order to select those locations for enhanced visual processing. Second, the shape of an object’s stereoscopic contour can be extracted independently from the shape of its luminance contour [15], so stereopsis could provide supplementary shape information by defining the object’s outline (bounding contour). Once the bounding contour has been extracted it could be used directly as a cue to the object’s identity; it could also be used to further restrict which part of the visual field contains the relevant 2D pattern information.

**Fig 1:**
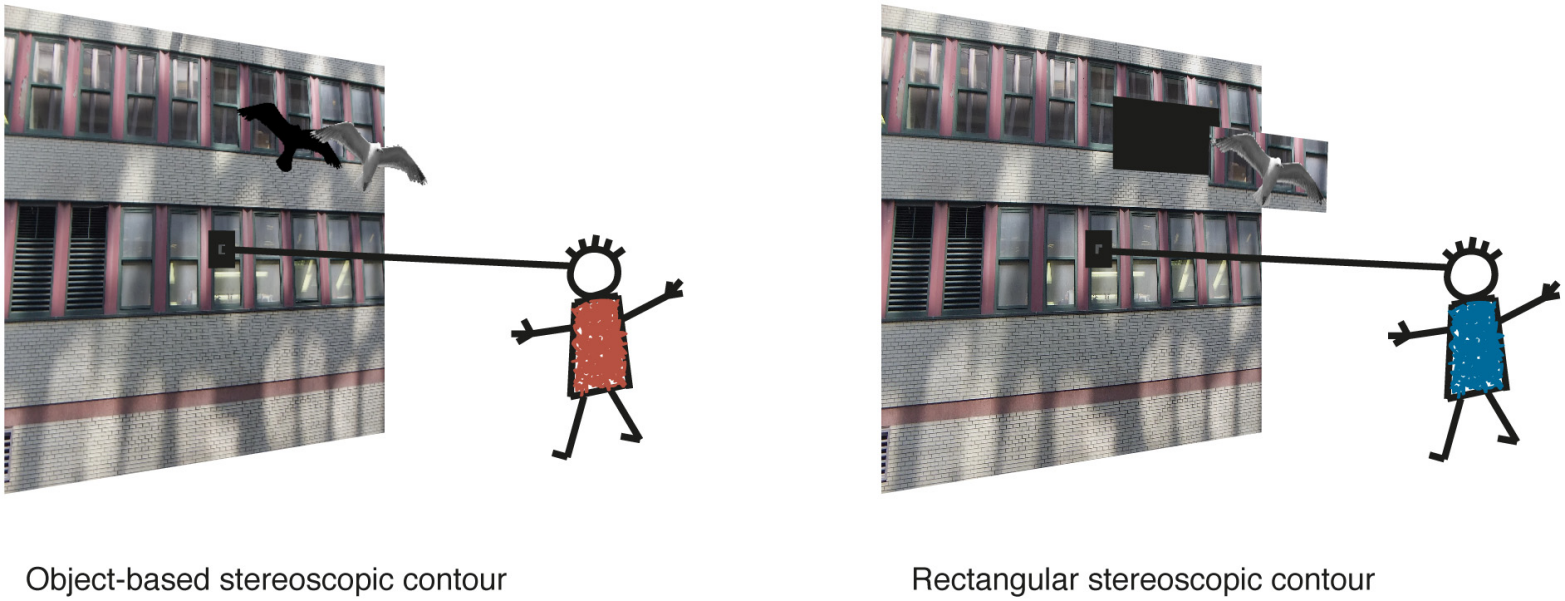
Appearance of the stimulus in the object-based (left) and rectangular (right) stereoscopic contour conditions. The target has the same disparity in both cases. In the first case the stereoscopic contour matches the shape of the luminance contour, but not in the second case.

These two mechanisms, orienting of attention and extraction of stereoscopic contours, are not mutually exclusive. Both could contribute to the recognition of an object in a natural scene. By manipulating the shape of the stereoscopic contour independent of the disparity of the target relative to the background, these two effects can be teased apart. We manipulated the background in the local region behind the target so that it would have either the the same disparity as the rest of the background or the same disparity as the target (Fig 1). In both cases the target object had a given disparity relative to the background, however the stereoscopic contour was coincident with the luminance contour of the target in the first case but not in the second.

There were thus four plausible outcomes. Disparity could (1) have no effect on the recognition of objects, (2) improve recognition only when the shape of the stereoscopic contour matched the shape of the luminance contour, (3) improve recognition equally regardless of the shape of the stereoscopic contour, or (4) improve recognition for both types of stereoscopic contour, while having a stronger effect for one type of stereoscopic contour than the other. Our results show that disparity improved recognition of objects regardless of the shape of the stereoscopic contour at the shortest display durations. However at longer display durations, recognition was higher when the shape of the stereoscopic contour matched the shape of the luminance contour. Therefore stereopsis appears to be doubly useful for recognizing objects in natural scenes.

## Experiment 1

### Methods

#### Observers

Observers were 15 students and faculty at the SUNY College of Optometry (mean age 24.4 ± 2.6 *SD*). All observers had normal or corrected-to-normal vision and a stereoacuity of 20 arcsec or better as measured with the Randot stereoacuity test (Precision Vision, La Salle, IL, USA). One additional observer participated in the experiment but was not included in the analysis [16].

#### Ethics statement

The study was approved by the SUNY College of Optometry Institutional Review Board and all observers gave written consent before participating in the experiment.

#### Stimuli and materials

Fifty-six frontal (en face) stereo pictures of building façades were taken in New York City using a stereo camera FinePix REAL 3D W3 (Fujifilm, Tokyo, Japan). Pictures were taken from across the street, at approximately 15 meters distance since the width of Manhattan streets is fixed at 60 imperial system feet [17]. The façades are of many different shapes, colors and textures across the different pictures. The façades were not generally perfectly orthogonal to the camera axis so backgrounds were slightly slanted, so that the relative disparity of the target depended on its location within the background, especially in the 0-disparity condition. On half of trials the background was synoptic—the same image was displayed to both eyes. The background type (synoptic or stereo) had no significant effect on performance, moreover there were no interactions with other experimental factors (see S5 Notes). Since background type did not explain any of the results, data from the two background conditions were averaged during analysis.

Stimuli were displayed on a rear projection screen subtending 64x50 degrees at a distance of 200 cm from the observer, who was seated in a chair. Binocular viewing was obtained using shutter glasses 3DN-6100 (3D NOW, Tamarac, FL, USA). A fixation box (8 arcmin) was displayed at the center of the screen with a vergence demand of 0.23 degrees, corresponding to a viewing distance of 15 m, which was approximately the distance at which the pictures were taken assuming an interpupillary distance of 6.0 cm. Prior to the experiment, the disparity at the fixation location of each background picture was manually adjusted by the experimenters to match the disparity of the fixation mark.

Target objects were stereoscopically flat pictures of three common birds in New York City (pigeon, sparrow and seagull) and three common flying man-made objects (plane, helicopter and dirigible). These 6 possible targets were the same in all the trials. The luminance contour of the targets were estimated using a thresholding technique, then manually adjusted by eye. Because the targets were the same throughout the experiment they would have become easily recognizable based on their color only. To minimize the use of this strategy we kept the colors of the different targets similar by converting them to gray scale images. The gray levels were then normalized to have the same mean luminance and contrast as defined by the variance of the luminance values in the target image (i.e. the first and second moments of the pixels’ gray level distributions were normalized). The background images were not converted to gray scale. Large differences in performance were found between the different target objects (see S3 Notes), but the effects described in the Results were observed for every target object.

The target object pictures subtended 10 degrees in their largest dimension—height or width—and were displayed at one of 16 equally spaced locations at 12 degrees of eccentricity from fixation. We used eccentric targets to avoid ceiling effects as central targets were recognized at very high rates under all conditions. The typical location of the blind spot in the visual field is 1.5 degrees below the horizontal meridian at 16 degrees of eccentricity on the temporal side, and its typical size is 10 degrees vertically by 7 degrees horizontally [18]. Therefore the target object slightly overlapped the blind spot for 2 of the outermost right and 2 of the outermost left target locations. However performance was not different at these locations, probably because most of the target was outside the blind spot (see S4 Notes).

Disparity and display duration were varied to explore the parameter space of a possible stereoscopic contour effect. Targets were displayed with one of five possible crossed disparities relative to fixation (0, 15, 30, 60 or 120 arcmin), and stimuli were displayed for one of four possible display durations (33, 67, 100 or 133 ms). Disparities followed a ratio scale because disparity thresholds follow Weber’s law to first approximation [19–21]. The left and right borders of the projection screen itself provided vertical contours at the viewing distance of 200 cm. The target had a disparity relative to these borders, in addition to its disparity relative to the background (at simulated 15 m). However, the target and borders were separated by 15 deg or more of visual angle, and small relative disparities are not robustly extracted across such large distances without changes of fixation [22–24], so this disparity is unlikely to have been important for our task.

**Fig 2:**
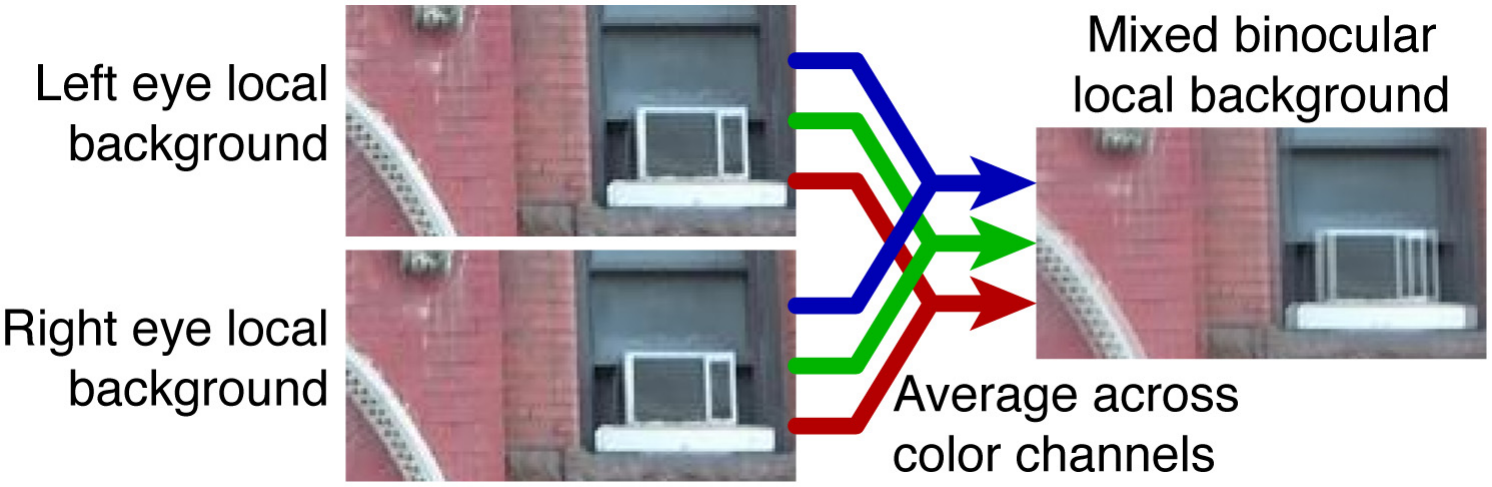
Method used to create the rectangular local backgrounds. The rectangular local backgrounds behind the target object in each eye were averaged across color channels pixel-by-pixel, after the rectangles were horizontally displaced relative to each other by an amount equal to the target’s disparity.

**Fig 3:**
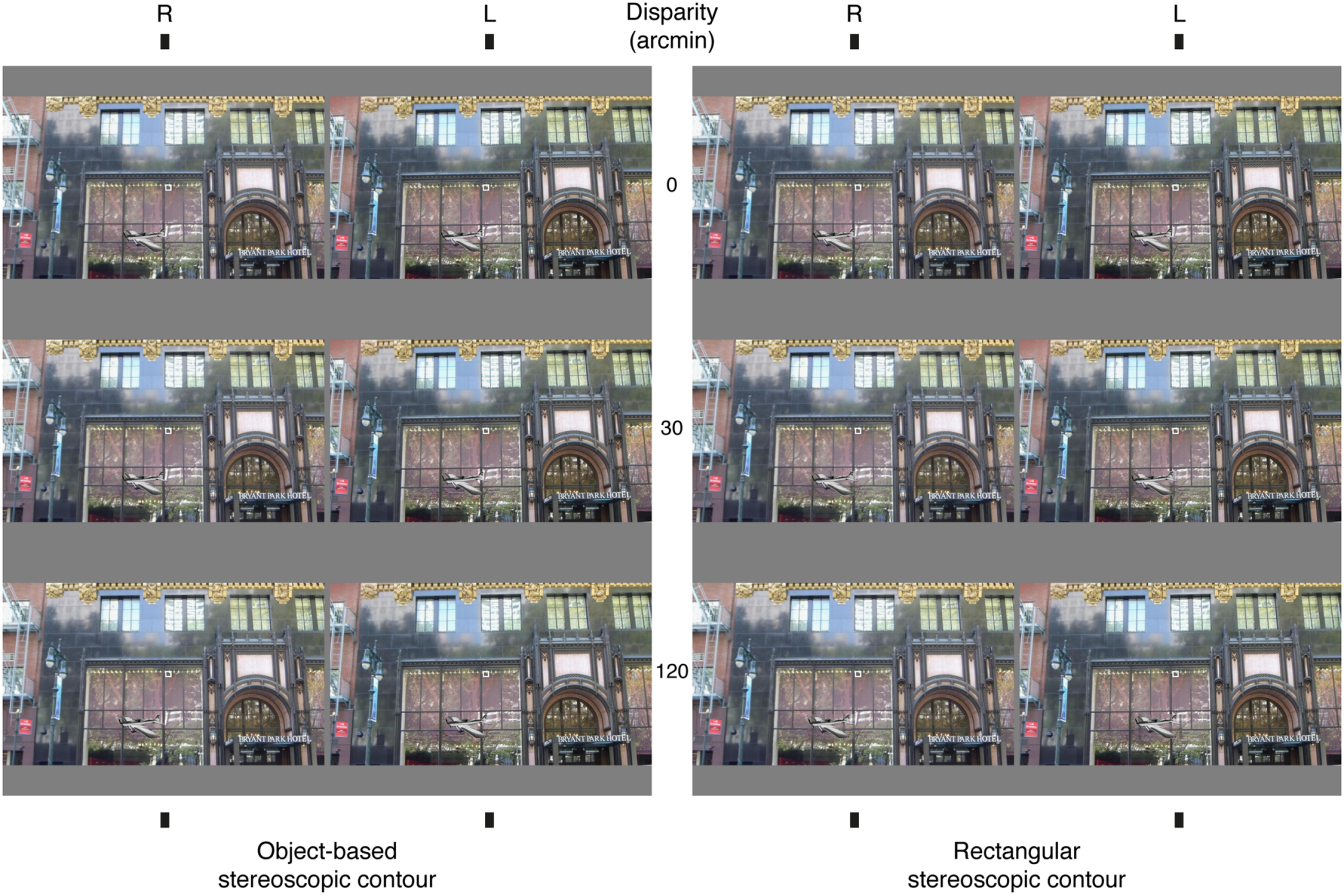
Examples of stimuli at 3 different disparities (0, 30 and 120 arcmin) and with the 2 types of stereoscopic contour conditions (object-based and rectangular), against a binocular background (synoptic background not shown). Arranged for crossed fusion. See Methods for stimulus dimensions, viewing distance, etc.

On half the trials the local background behind the target in each eye (a rectangle of the same horizontal and vertical size as the target object) was averaged across each color channel at the same disparity as the target, so that it became a blended mixture of the left and right local backgrounds (Fig 2). This procedure allowed us to present the local background at the same horizontal disparity as the target while minimizing disruption of the stimulus by avoiding the empty monocular zones and full-contrast double image features introduced when disparities are manipulated. The target appeared embedded on a rectangular local background. Fig 1 represents how the stimuli appeared to the subjects in the 2 stereoscopic contour conditions. Fig 3 shows different examples of stimuli.

Masks were presented immediately after the stimulus. Each mask consisted of 200 random geometrical shapes. Each shape had between 6 and 12 sides, was approximately the same size as the target, had a color that was randomly sampled from the background and target pictures, and had a disparity randomly selected in the range of possible disparities of the target. The power spectrum of such a mask roughly matches the power spectrum of natural pictures, *p = f* ^*-2*^ [25,26].

#### Procedure

The different factors of the experiment are described in Table 1. The factors *display duration* (33, 67, 100 or 133 ms), *disparity* (0, 15, 30, 60, 120 arcmin), *stereoscopic contour* (object-based or rectangular), *background condition* (synoptic or binocular) and *target object* (1 of the 6 target images) were crossed. The factors *target location* and *background picture* were pseudo-randomized in a partial Latin square design so that the same target object would not always be displayed over the same background or at the same location. Each session contained 960 trials (two trials per combination of *target image*, *duration*, *disparity*, *contour type* and *background type*). The order of the trials was completely randomized.

**Table 1:** Experimental factors.

Factor	Values	Design
**Display duration**	4 (33, 67, 100,133ms)	Crossed
**Disparity**	5 (0, 15, 30, 60, 120arcmin)	Crossed
**Stereoscopic contour**	2 (object-based, rectangular)	Crossed
**Background type**	2 (synoptic, binocular)	Crossed
**Target object**	6 (see text)	Crossed
**Location**	16 (equally spaced on a circle)	Pseudo-randomized
**Background picture**	56 stereo-pair images	Pseudo-randomized

The factors of display duration, disparity, stereoscopic contour, background type, and target object were fully crossed. Factors of location and background picture were pseudo-randomized (partial Latin square design).

**Fig 4:**
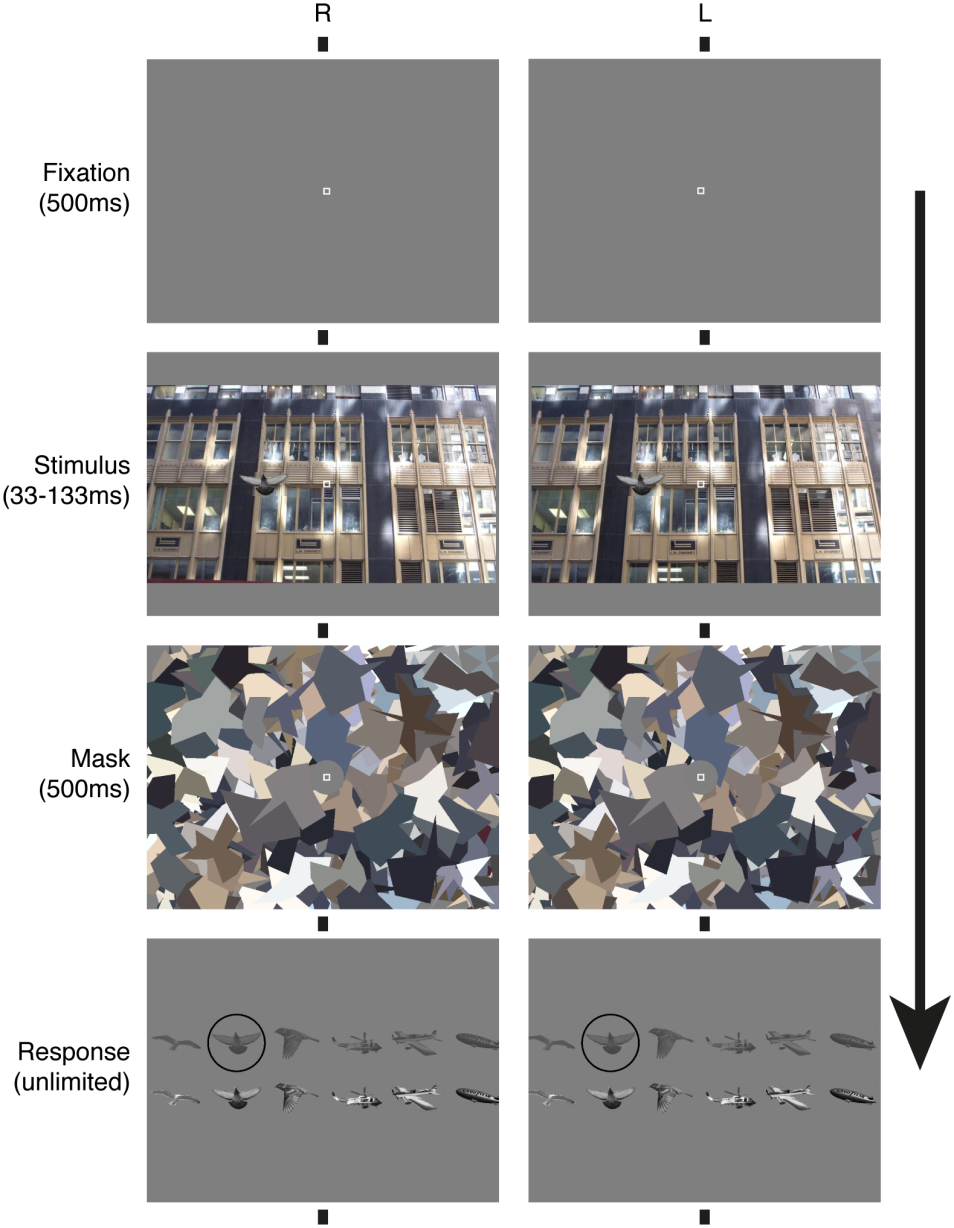
Time course of a trial: observers fixated for 500 ms. The stimulus was then displayed for 33 to 133 ms and immediately followed by a 500 ms mask. Finally the observers had to report which target object was contained in the stimulus and how confident they were in their answer by moving the black circle cursor. Here the black cursor indicates that the pigeon target was displayed and that the observer is not confident in his/her answer. The figure can be cross-fused.

Fig 4 represents a trial sequence. After a 500 ms fixation, the stimulus was displayed for a duration between 33 and 133 ms and then masked for 500 ms. Then the 6 possible targets were displayed on the bottom row of a two-row display. On the row above, the same targets were displayed again with half luminance. The task of the observer was twofold. They had to report which target was contained in the stimulus and also to report the confidence in their answer by selecting the dark item on the upper row for low confidence or the bright item on the lower row for high confidence. Selection between the 12 response items was done by moving a black circle cursor with the keypad arrows. Observers were explicitly instructed to find their own criterion between high and low confidence. The next trial started 500 ms after the observer validated their answer by pressing the keypad return key. The observers had unlimited time to respond and the initial location of the black cursor was randomized.

### Results and Discussion

**Fig 5:**
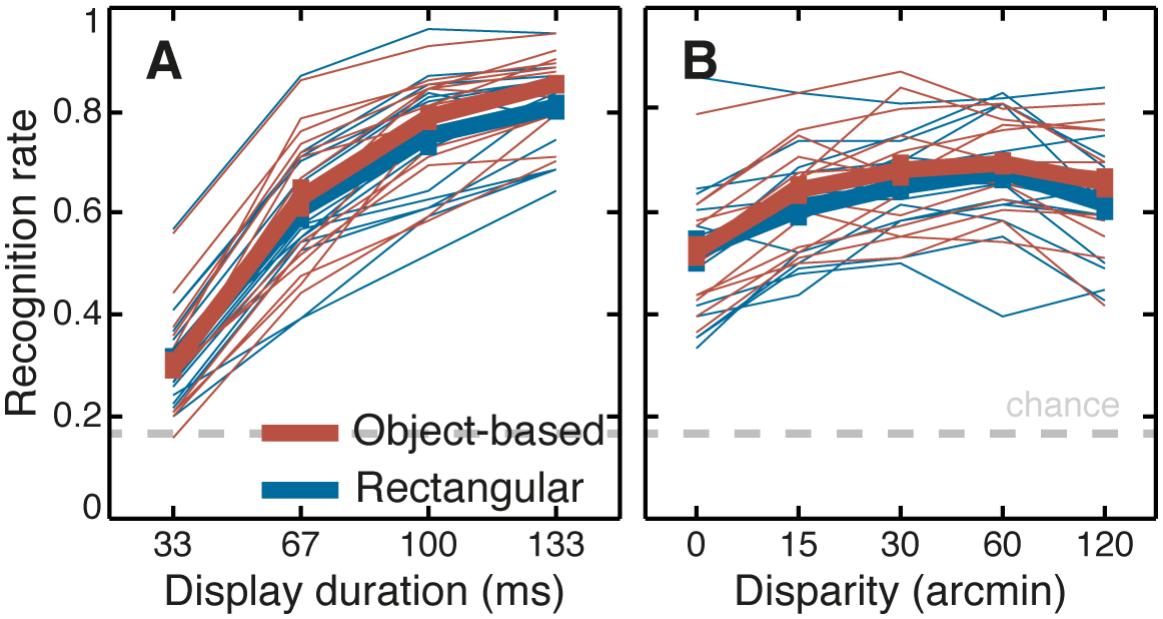
Results of Experiment 1. A: Recognition rate as a function of display duration. B: Recognition rate as a function of disparity. Red lines are object-based stereoscopic contours and blue lines are rectangular stereoscopic contours. Thick lines are population average with standard errors, thin lines are individual observers. The gray dashed line plots the recognition rate for chance performance.

Fig 5 shows recognition rate, i.e. the proportion of correct responses, as a function of display duration (A), disparity (B) and as a function of the stereoscopic contour condition (red and blue lines). All of the following statistical analyses were made on recognition rates transformed into z-scores and all t-tests were two-tailed. Z-scores were used to linearize ratios in an arbitrary unit that is linearly related to *d’* in Signal Detection Theory [27–30]. The pattern of confidence judgments was strikingly similar to recognition and the results of statistical analyses were identical to recognition (see S1 Notes).

**Fig 6:**
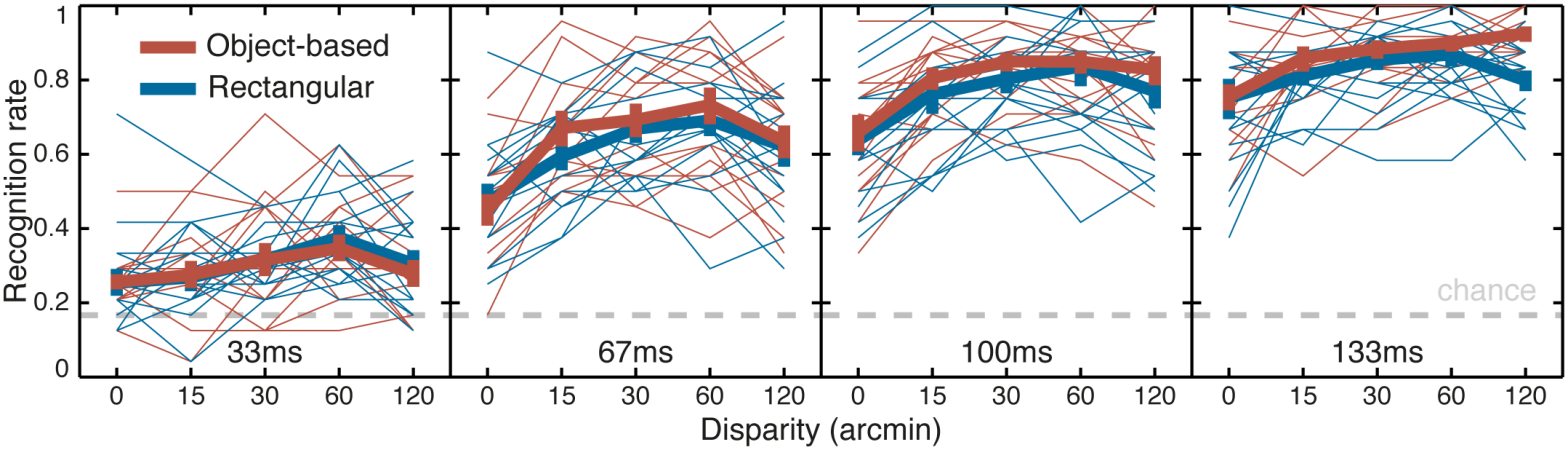
Results of Experiment 1. Lattice plot of the recognition rate as a function of disparity (in abscise) and display duration (from left to right: 33, 67, 100 and 133ms) for the object-based stereoscopic contour condition (red lines) and rectangular stereoscopic contour condition (blue lines). Thick lines are population mean and standard errors, thin lines are individual observers. The gray dashed line plots the recognition rate for chance performance.

The effect of *presence of disparity* was assessed by comparing the z-transformed recognition rate for the (combined) nonzero disparity conditions to the 0-disparity condition for each individual. The mean recognition rate increased from 52.1% to 64.8% with the addition of disparity to the display, for a mean improvement of 12.7 ± 1.7% (*SE*). The significance of this difference, and its interaction with display duration, was assessed in a two-way repeated measures ANOVA with factors of *presence of disparity* (2 levels) and *display duration* (4 levels). *Stereoscopic contour* was not a factor because it is undefined in the 0-disparity condition. Both main factors were significant: F(3,42) = 415, p<0.0001; F(1,14) = 50.7, p<0.0001 for *display duration* and *presence of disparity*, respectively. In addition the interaction between these two factors was significant, F(3,42) = 5.69, p = 0.0023. Fig 6 shows the nature of this interaction: disparity provided less benefit at the shortest display duration (33 ms) than at longer display durations. Nevertheless, a separate, post-hoc paired t-test confirmed that *presence of disparity* had a significant effect even at the shortest display duration of 33 ms, where it increased the mean recognition rate from 26.4% to 32.2% for a mean difference of 5.7 ± 2.2% *(SE)*, t(14) = 2.58, p = 0.022.

The 0-disparity condition was excluded from further analyses, including ANOVAs and t-tests. To better characterize the effect of disparity, the z-transformed recognition rates were fitted with polynomial functions for each observer and t-tests were made on the distributions of fit parameters. The main effect of disparity across nonzero values was assessed by fitting a parabola (second order polynomial, *y = ax* ^*2*^ *+bx+c*) on the z-transformed recognition rates as a function of log disparity for each individual. The second order polynomial fit parameter (curvature) was significantly inferior to 0, t(14) = 5.24, p = 0.0001; indicating that the curve was concave down. The peak value (vertex) of the parabolas had a mean value of 51.3 ± 1.1 (*SE*) arcmin, with the exclusion of one observer who did not have a negative second-order polynomial fit parameter. The peak value was significantly higher than zero, t(13) = 30.6, p<0.0001; but significantly lower than 120 arcmin, t(13) = 6.60, p<0.0001, the maximum disparity value of the target objects, indicating that recognition did decrease at the highest disparity value, i.e. the mean curve was concave and the peak value was contained in the range of the displayed disparities.

On average, recognition rates were higher in the object-based stereoscopic contour condition than in the rectangular stereoscopic contour condition. The magnitude of this benefit was small but statistically significant: recognition rates were 60.9% and 63.5% in the two contour conditions, respectively, for a mean benefit of 2.61 ± 0.60% (*SE*), t(14) = 6.52, p<0.0001. For comparison, recall that the benefit of disparity *per se*—the benefit of nonzero-disparity conditions over the 0-disparity condition—was 13%.

Fig 5 (A) shows a small but significant interaction between stereoscopic contour and display duration, with the object-based contour becoming relatively more effective as display duration increases. We measured this interaction by fitting a straight line to the z-transformed recognition rates for each contour condition and individual. The difference between the slopes of the fitted lines in the 2 stereoscopic contour conditions was 2.53 ± 0.64 (*SE*) z-score units per second (0.042 per display frame), t(14) = 3.95, p = 0.0015.

Fig 6 shows the interaction between disparity, display duration and stereoscopic contour in a lattice plot. The interaction between stereoscopic contour condition and display duration is clear with overlapping curves at the shortest display duration and completely separated curves—except at 0-disparity where the stereoscopic contour is undefined—at the longest display duration.

The data plotted in Figs 5 and 6 might suggest additional interactions. Unlike the effect of duration, however, the effect of disparity was not monotonically increasing, and there was no similar rational basis for comparing slopes to determine interactions. A given interaction could occur in any of several ways, so to avoid missing an interaction, a three-way repeated measures ANOVA with *durations*, *disparities*, *contours* as factors was run on z-transformed recognition rates for the nonzero-disparity stimuli. The results of the ANOVA were consistent with the previous analyses, showing significant effects of the 3 main factors, F(3,42) = 399, p<0.0001; F(3,42) = 5.55, p = 0.0027; F(1,14) = 20.6, p = 0.0005; for duration, disparity and contour, respectively. Also, in accord with the previous analyses the interaction between stereoscopic contour and display duration was significant, F(3,42) = 4.40, p = 0.0088, but not the interaction between disparity and stereoscopic contour, F(3,42) = 1.35, p = 0.27. Finally the interaction between duration and disparity and the 3-way interaction between duration, disparity and contour, were not significant, F(9,126) = 0.254, p = 0.99; F(9,126) = 1.09, p = 0.38; respectively.

In summary, in Experiment 1 performance was higher for targets with disparity. Performance increased with display duration and it also increased with disparity, peaking at about 1 degree of disparity before decreasing at higher values. There was a significant interaction between display duration and stereoscopic contour, with an advantage for object-based contours that increased with display duration. These results, which were replicated in the confidence ratings (see S1 Notes), confirmed that putting a target object into crossed disparity relative to its background made it easier to recognize, and that given enough viewing time (more than 33 ms) the stereoscopic contour provided additional information that was useful for recognition.

## Experiment 2

Experiment 1 showed that object recognition was better for object-based than rectangular stereoscopic contours. The rectangular-contour condition in Experiment 1 was designed so as to minimally change other aspects of the stimulus, by averaging the local backgrounds behind the target. To rule out the possibility that this image manipulation made objects harder to recognize, we ran the same experiment while the observer wore an eyepatch. Since the stereoscopic contour was no longer present, any surviving difference between the two stereoscopic contour conditions shows the effect of the local background *per se*. The results showed that the local background did not have any effect on performance, confirming that the effect of stereoscopic contour type in Experiment 1 was due to the stereoscopic contour and not the blended local background.

### Methods

#### Observers

Power calculation through Monte-Carlo simulation on the results of Experiment 1 showed that the difference between the average z-score in the two stereoscopic contour conditions would be significant at the 0.05 level 83% of the time for 8 observers. Therefore 8 observers ran Experiment 2 (mean age 24.2 ± 1.5 *SD*). One of the observers had also participated in Experiment 1.

#### Ethics statement

The study was approved by the SUNY College of Optometry Institutional Review Board and all observers gave written consent before participating in the experiment.

#### Stimuli, materials and procedure

Experiment 2 was identical to Experiment 1 except that observers wore an eye patch over their non-dominant eye.

### Results and discussion

**Fig 7:**
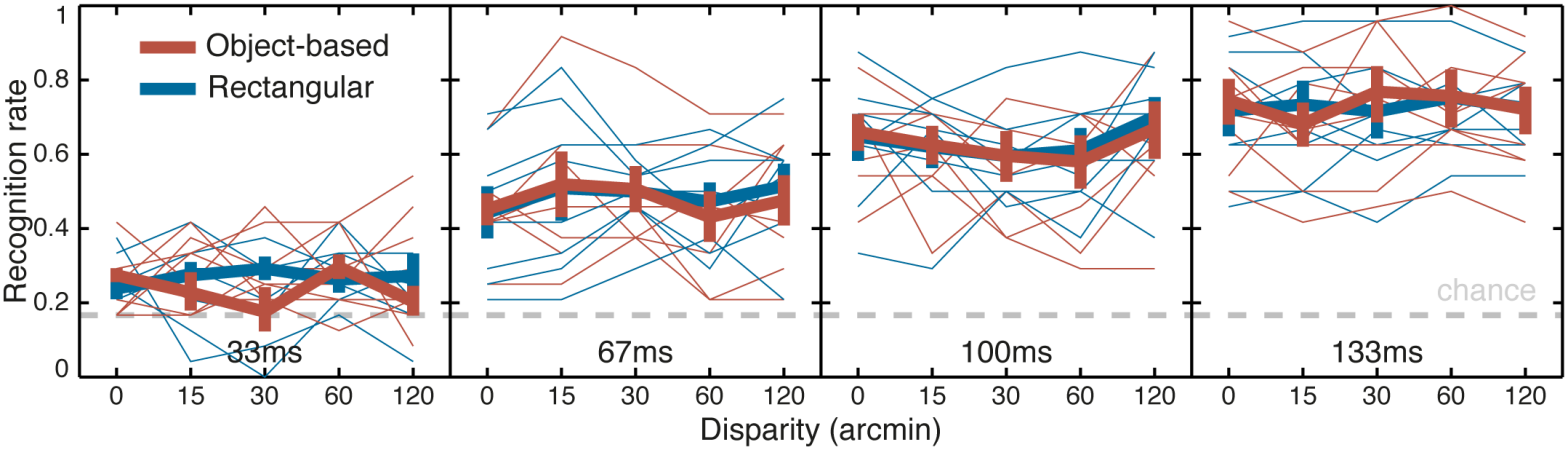
Results of Experiment 2, a control in which observers wore an eye patch. Disparity affected the stimulus because the displacement between the overlapped images in the local background of the rectangular-stereoscopic contour condition was equal to the disparity. Lattice plot of the recognition rate as a function of disparity (in abscise) and display duration (from left to right: 33, 67, 100 and 133ms) for the object-based stereoscopic contour condition (red lines) and rectangular stereoscopic contour condition (blue lines). Thick lines are population mean and standard errors, thin lines are individual observers. The gray dashed line plots the recognition rate for chance performance.

Fig 7 shows performance in Experiment 2. Fig 7 is similar to Fig 6 except that observers wore an eye patch during the experiment. As in experiment 1, the pattern of confidence judgments was similar to recognition and statistical analyses were identical (see S1 Notes).

Unsurprisingly the disparity of the target object ceased to modulate performance. Importantly, there was no effect of the stereoscopic contour condition on performance: performance was better in the rectangular contour condition by 1.3 ± 1.4% (*SE*), (i.e. the trend was in the opposite direction from the finding in Experiment 1) and this difference was not statistically significant, t(7) = 0.89, p = 0.40.

A three-way repeated measures ANOVA found a significant effect of display duration, F(3,21) = 109, p<0.0001; but no effect of either disparity or stereoscopic contour. None of the second and third order interactions were significant. There was no significant cost of using only one eye in Experiment 2 as compared to the zero-disparity condition of Experiment 1. Overall, average performance across all conditions was significantly better in Experiment 1 (62% correct) than in Experiment 2 (52% correct), t(21) = 2.25, p = 0.036, but most of this benefit came from the presence of nonzero disparity in Experiment 1: the recognition rates for the 0-disparity condition were similar in the two experiments (52.1% vs. 51.5% correct, respectively), t(21) = 0.16, p = 0.87.

In summary in Experiment 2, recognition was modulated by the display duration only. Neither the target disparity nor the stereoscopic contour modulated performance, which demonstrates that the mixed local backgrounds used in the rectangular-stereoscopic contour condition of Experiment 1 were not responsible for the lower recognition rate observed for that condition as compared to the object-based contour condition.

## Experiment 3

Experiment 1 showed that recognition of objects is improved when the shape of the stereoscopic contour matches the shape of the luminance contour of the target. To confirm the plausibility of a facilitating object-shaped contour effect under the conditions of our experiment, we tested whether the objects could be discriminated based on the shape of the stereoscopic contour alone. Thus in this experiment the target objects were displayed in a random-dots stereogram (RDS) and were therefore invisible monocularly [15]. Recognition of the target was mediated only by the shape of its stereoscopic contour.

### Methods

#### Observers

Observers were 8 students and faculty at the SUNY College of Optometry (mean age 31.3 +/- 8.5 *SD*), all with normal or corrected-to-normal vision and a stereoacuity of 20 arcsec or better (Randot).

#### Ethics statement

The study was approved by the SUNY College of Optometry Institutional Review Board and all observers gave written consent before participating in the experiment.

#### Stimuli and materials

The shapes of the same six target objects as in Experiments 1 and 2 were displayed as RDS. For each trial a dense background random dots texture consisting of 2x2 monitor pixel elements was generated. Each element had a luminance value sampled uniformly from the entire range of luminance allowed by the projector. The background was displayed at the same uncrossed disparity as background images in Experiment 1. In each background texture, elements corresponding to the target object for the trial were horizontally displaced by an amount corresponding to one of the 4 nonzero disparities in Experiment 1 (15, 30, 60 and 120 arcmin), half of the displacement occurring in each eye. Because the effect of the shape of the stereoscopic contour increased with disparity, only the 2 longest display durations in Experiment 1 were used in this experiment (100 and 133 ms). The target object was displayed at one of 8 equally spaced target locations in the stimulus. The masks were generated similarly to Experiments 1 and 2. The mask colors were randomly sampled from the stimulus, so they were gray. Parameters and apparatus in this experiment were otherwise similar to Experiment 1. All the experimental factors were crossed including location, resulting in 384 types of trial (2 display durations, 4 disparities, 6 target objects and 8 target locations), each of which was shown once in each session.

#### Procedure

After a 1000 ms fixation period, the stimulus was displayed for 100 or 133 ms and then was masked. Observers then reported which target object was contained in the stimulus. As in Experiments 1 and 2 the observer had to move a black circle cursor to choose one of the 6 possible target objects. However the target objects were black against a gray background in a single row, and confidence judgments were not recorded.

### Results and discussion

**Fig 8:**
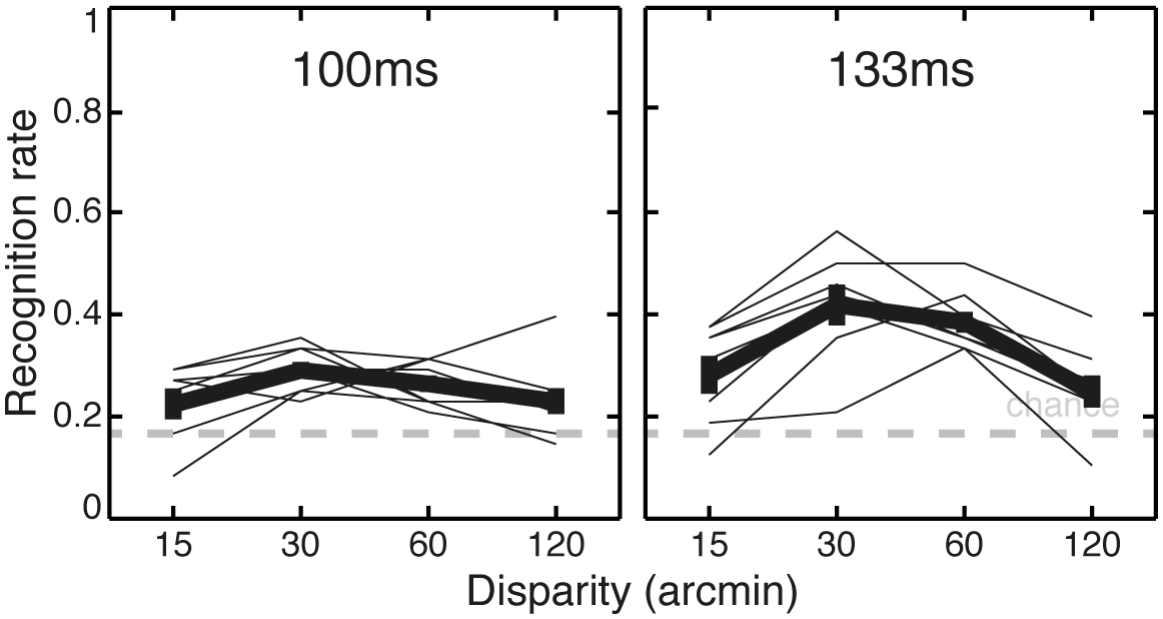
Results of Experiment 3. Recognition rate as a function of disparity (in abscise) for two display durations (left 100ms and right 133ms). Thick lines are population mean and standard errors, thin lines are individual observers. The gray dashed line plots the recognition rate for chance performance.

Fig 8 shows recognition rate as a function of display duration and disparity. Observers performed better than chance (16.7%): the percentage of correct answers across all conditions was 29.7 ± 1.7% (*SE*), which was significantly higher than chance in a binomial test, p≤0.002, for each of the 8 observers separately. A two-way repeated measures ANOVA with duration and disparity on z-transformed recognition rates found that the main effects of duration and disparity were both significant, F(1,7) = 11.5, p = 0.012; F(3,21) = 11.7, p = 0.0001; respectively. The interaction between display duration and disparity did not reach significance, F(3,21) = 2.26, p = 0.11.

Recognition increased with display duration from 25.6% to 33.8%, t(7) = 3.73, p = 0.007. Recognition peaked at a shorter disparity value than in Experiment 1. To quantify this effect the individual z-transformed recognition rates were fitted with second order polynomials as a function of log disparity. The peak value was 39.3 ± 1.1 (*SE*) arcmin, which was significantly higher than the lowest target disparity value of 15 arcmin and lower than the highest disparity value of 120 arcmin, t(7) = 10.8, p<0.0001, t(7) = 12.5, p<0.0001; respectively. The mean peak value in this experiment was less than the mean peak value of 51.3 ± 1.1 (*SE*) arcmin in Experiment 1, however this difference between the two experiments was not significant in a two-sample (unpaired) t-test, t(20) = 1.45, p = 0.16.

In summary this experiment showed that the stereoscopic shape alone was sufficient for observers to recognize the target objects, although performance was low in absolute terms even at the longest display duration.

## General Discussion

Does stereopsis normally contribute to the recognition of objects in natural scenes? We found that for at least one class of natural scenes making targets stereoscopically closer than their backgrounds caused an increase in recognition rate at all of the display durations we tested. Because we used naturalistic images, it is reasonable to conclude that this benefit extends to natural viewing situations. Immediately after a head turn or large saccade, for example, a person who has stereo vision should be able to recognize near objects with greater speed and accuracy than if that person did not have stereo vision.

In our experiments disparity had a facilitating effect across a large range of values, with a peak at about 1 degree of relative disparity between the target and the background and still present for targets with 2 degrees of disparity relative to the background. Most (approximately 75%) of the benefit from disparity in our experiments did not depend on whether the target object’s disparity contour coincided with its luminance contour. This fact suggests that most of the facilitation effect resulted from the orienting of attention to the 2D or 3D location of the target. Disparity “pops out” in search tasks, and disparity presumably attracted attention to the location of the target [12,14,31–34]. This orienting of attention evidently occurred sufficiently quickly to facilitate recognition, based on the object’s 2D pattern, before the pattern faded from iconic memory [35].

In addition, however, average performance was higher when the shape of the stereoscopic contour matched the shape of the target. This benefit was not present at the shortest display duration of 33 ms but emerged at longer display durations. Experiment 2 demonstrated that in absence of disparity, the benefit of the contour disappeared, which ruled out artifactual explanations based on changes to the image in the local background of the target. Experiment 3 demonstrated that observers were able to recognize the target objects at levels above chance in the absence of luminance patterns and luminance contours despite the lower spatio-temporal resolution of the stereoscopic system compared to the luminance system [36]. The low recognition rate in Experiment 3 can be seen as a minimum performance for processing stereoscopic contours, since stereopsis is generally facilitated when disparities are carried by luminance contours [37,38].

The fact that stereo contours can be extracted under the relatively impoverished conditions of experiment 3 does not *prove* that the stereo contours in experiment 1 contributed shape information that facilitated recognition. For example, small shapes or jagged contours might be better at attracting attention than larger rectangles; if so, additional attention might account for the additional benefit seen for objects with stereo-defined contours in experiment 1. However, this account seems unlikely because in experiment 1 the benefit conferred by the object-based contour was delayed relative to the benefit conferred by disparity *per se*. It seems likely that the shape of the contour contributed to recognition in both experiments, not just experiment 3.

Any attentional resources that were attracted to the target presumably improved not only the extraction of 2D pattern information, but also the extraction of the target’s stereoscopic contour. The exact mechanism(s) by which a stereoscopic contour could contribute to the recognition of a luminance-defined object (as in experiment 1) remain to be identified. One possibility is that the bounding contour of the object is processed twice, from both the 2D luminance pattern and from the stereoscopic contours, in which case the stereoscopic contour would help through probability summation. Another possibility is that the stereoscopic contour contributes indirectly to recognition, for example by contributing to the segmentation process [39–41], by providing spatial restrictions that contribute to object-based attention [42,43] or by releasing simultaneous contrast masking from the background [44].

The importance of stereoscopic contours for object recognition under natural conditions must of course depend greatly on details of those conditions. In our experiments, the background images were relatively flat fronto-parallel surfaces, in which case the center-surround saliency effect of stereopsis was at its strongest [13]; also, targets were presented in eccentric vision. In real life a visual scene is often populated by many objects at different distances, which would reduce the disparity-based salience of the target and thus its ability to attract attention; and targets are often fixated. The target’s stereoscopic contour could potentially become *relatively* more important under these conditions because central targets would be more salient, are already attended, and their stereo-defined contours are more easily extracted.

One cannot assume that experiments using reduced, artificial stimuli such as Gabor patches will account for performance under natural conditions [45]. Many signals besides disparity are important for recognition. In previous studies, stereopsis did not improve the recognition of whole natural scenes even though it improved recognition of artificial scenes [26], and within-object disparity was sometimes effective only for recognizing artificial stimuli [11]. Additional work with synthetic stimuli will be necessary to study the underlying mechanisms, but the significance of our result is that using a common class of naturalistic binocular visual scenes, stereopsis played a role to help identify objects quickly and accurately.

An unnatural feature of our stimuli was their stereoscopic flatness, which allowed us to embed them in a flat rectangle. If the visual system could determine the targets’ flatness reliably, it would constitute a conflicting 3D shape cue, and to the extent that stereo 3D shape contributes to object recognition, it might cause an artifactual decrease in recognition performance. Fortunately this scenario is implausible. First, even when targets are near in distance, presented in central vision, and temporally persistent, internal disparity structure is not well utilized for recognition (see Introduction). By contrast, our targets were far, eccentric, and brief. Second, target flatness would have been difficult for the visual system to determine. For example, at the far display distance of 15m, the target’s width of 10 deg corresponds to a linear width of 2.6m; if it were half as deep (1.3m) it would contain 1.2 arcmin of relative disparity. Similarly, at all simulated distances, the within-object disparity would be close to threshold, or below [19–21], so that variation in disparity across the object could not be a useful cue—or provide a cue conflict—even if present.

Intriguingly, observers’ confidence judgments were strikingly similar to their recognition rates. Observers had remarkable insight into how well they were able to do the task. One explanation is that performance may have been based on a visual memory of the stimulus, with observers being able to judge how well formed their representation was on a given trial. A well-formed representation would lead both to better performance and, insofar as confidence reflects the uncertainty in an answer, to higher confidence [46,47].

Another interesting aspect of the data is the large range over which the facilitation effect of stereopsis occurred. The facilitation effect peaked at about 1 degree of relative disparity between the target and the background, and even though recognition decreased at higher disparity values, it remained higher for targets with 2 degrees of disparity than for targets with zero disparity. The decrease in performance at high disparities is likely due to diplopia, or to the same factors that cause diplopia [20,48,49].

Finally, in these experiments the target objects always had a crossed disparity relative to the background [16]. We cannot conclude that all disparity contrasts attract attention. It is possible that only objects with crossed disparities, closer to the observer than the background behind them, can attract attention, which would be consistent with previously published studies on the orienting of attention in depth [33,50–52].

In conclusion this series of experiments shows that stereopsis is doubly useful for the recognition of objects in naturalistic scenes. First, disparity attracts attention to the spatial location of the object. Second, it provides supplementary information about the shape of the object. Thus, stereopsis has roles to play during mid-level visual processing. In particular, the usefulness of binocular vision is not limited to the perception of accurate stereoscopic depth. Binocular disparities play a role very early during visual processing by contributing to the orientating of attention to the relevant part of a visual scene, and probably also the formation of object representations through the shapes of their stereoscopically defined contours.

## Supporting Information

S1 Data Data of experiment 1. (TXT)

S2 Data Data of experiment 2. (TXT)

S3 Data Data of experiment 3. (TXT)

S1 Notes Confidence judgments. (PDF)

S2 Notes Camera artifacts. (PDF)

S3 Notes Effect of the target object. (PDF)

S4 Notes Effect of the target’s location. (PDF)

S5 Notes Effect of background type. (PDF)

S6 Notes Effect of disparity sign. (PDF)
