# Supplementary material for: Stereoscopic Offset Makes Objects Easier to Recognize
Source: PLoS One. 2015 Jun 16;10(6):e0129101. doi: 10.1371/journal.pone.0129101 (PMC4469586; doi:10.1371/journal.pone.0129101)
Supplement: S1 Notes — (PDF) [file pone.0129101.s004.pdf]

S1 Notes. Confidence judgments.

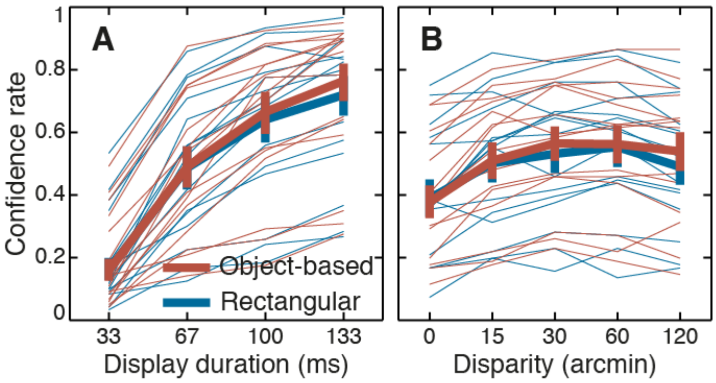

S1 Fig. Results of Experiment 1. A: Confidence rate as a function of display duration. B: Confidence rate as a function of disparity. Red lines are object-based stereoscopic contours and blue lines are rectangular stereoscopic contours. Thick lines are population average with standard errors, thin lines are individual observers.

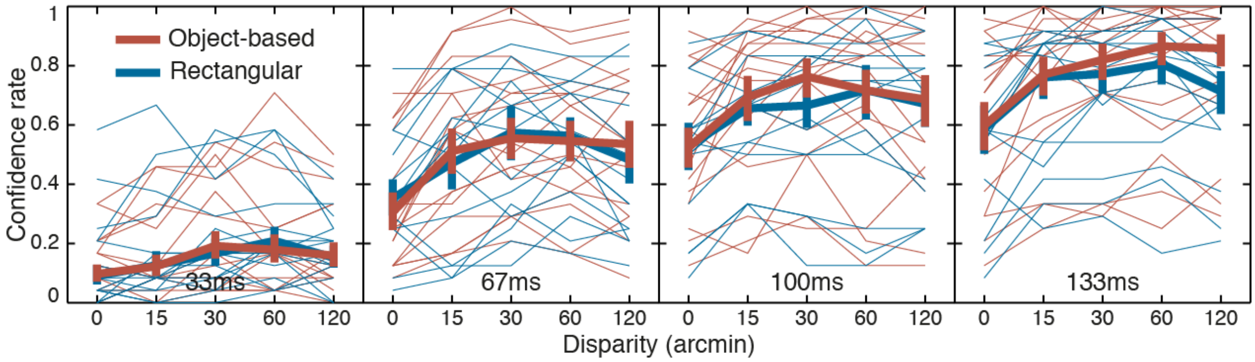

S2 Fig. Results of Experiment 1. Lattice plot of the confidence rate as a function of disparity (in abscise) and display duration (from left to right: 33, 67, 100 and 133ms) for the object-based stereoscopic contour condition (red lines) and rectangular stereoscopic contour condition (blue lines). Thick lines are population mean and standard errors, thin lines are individual observers.

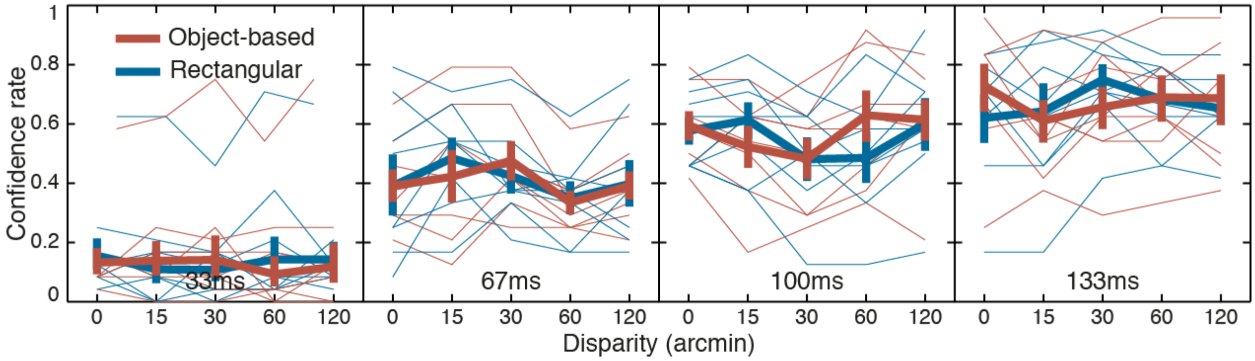

S3 Fig. Results of Experiment 2. Lattice plot of the confidence rate as a function of disparity (in abscise) and display duration (from left to right: 33, 67, 100 and 133ms) for the object-based stereoscopic contour condition (red lines) and rectangular stereoscopic contour condition (blue lines). Thick lines are population mean and standard errors, thin lines are individual observers.

Subjects indicated not only the target's identity, but also whether they had high or low confidence in their answer. S1 and S2 Figs. show confidence rates in Experiment 1, and S3 Fig. in Experiment 2. Confidence rates are the proportion of trials with a "high confidence" response. The pattern of data for confidence judgments was nearly identical to the pattern for recognition rates (compare to Figs. 5, 6 and 7). The correlation between confidence and recognition was computed for each observer across all of their (binary-outcome) trials, yielding a mean Pearson correlation (phi) coefficient of $r = 0.48 \pm 0.03$ (*SE*). Thus observers had remarkably good insight on a given trial as to the likelihood that their answer was correct. Table S1 describes the results of the ANOVA on z-transformed confidence rates from Experiment 1. The comparisons that were significant ($p < .05$) for confidence ratings (shown in the table) proved to be identical to those that were significant for recognition rates (main text) with the exception of the interaction between duration and stereoscopic contour, which did not reach significance ($p=0.098$).

S1 Table. Experiment 1.

Effect	F	d.f.	p-value	Significant
**Duration**	106	3,42	<0.0001	Yes
**Disparity**	9.19	3,42	<0.0001	Yes
**Contour**	8.29	1,14	0.012	Yes
**Duration & Disparity**	0.590	9,126	0.80	No
**Duration & Contour**	2.24	3,42	0.098	No
**Disparity & Contour**	1.24	3,42	0.31	No
**Duration & Disparity & Contour**	0.876	9,126	0.55	No

Results of the ANOVA on confidence judgments (fraction of "high confidence" answers). The ampersand indicates an interaction. The column "F" reports F-values, the column "d.f." reports degrees of freedom and the column "p-value" reports the p-values.

S2 Table describes the results of the ANOVA z-transformed confidence judgments in Experiment 2. The comparisons that were significant ($p < .05$) for confidence ratings (shown in the table) are identical to those that were significant for recognition rates (main text).

S2 Table. Experiment 2.

Effect	F	d.f.	p-value	Significant
**Duration**	36.7	3,21	<0.0001	Yes
**Disparity**	0.516	3,21	0.68	No
**Contour**	0.692	1,7	0.43	No
**Duration & Disparity**	1.61	9,63	0.13	No
**Duration & Contour**	0.353	3,21	0.79	No
**Disparity & Contour**	0.186	3,21	0.91	No
**Duration & Disparity & Contour**	1.96	9,63	0.059	No

Results of the ANOVA on confidence judgments. The ampersand indicates an interaction. The column "F" reports F-values, the column "d.f." reports degrees of freedom and the column "p-value" reports the p-values.
