# Supplementary material for: Stereoscopic Offset Makes Objects Easier to Recognize
Source: PLoS One. 2015 Jun 16;10(6):e0129101. doi: 10.1371/journal.pone.0129101 (PMC4469586; doi:10.1371/journal.pone.0129101)
Supplement: S2 Notes — (PDF) [file pone.0129101.s005.pdf]

S2 Notes. Camera artifacts.

The stereo camera (Fujifilm FinePix REAL 3D W3) used to take the background pictures is a commercial product that had two defects:

• The optical axes of the two lenses crossed at approximately 2.1 meter in front of the camera rather than being parallel. Displaying the pictures on a flat display screen therefore induced some perspective distortions. We estimated these distortions being negligible and unlikely to affect performance.

• Sensor rotation caused images to be excycloverged—clockwise rotation of the right image relative to the left image—by approximately 0.75 degrees. The cyclodisparity of the stimuli (see S4 Fig.) was not perceptually obvious and was not remarked upon by any observer, however it produced an artifactual relative disparity of the target to the background. The background had a small vertical disparity relative to the background to the left and right of fixation, uncrossed disparity at the top and crossed disparity at the bottom. Therefore in the 0-disparity condition, the targets had zero disparity relative to fixation but had an artifactual disparity relative to the background depending of their location in the stimulus. To estimate the effect of this artifact we used two different background conditions: in half the trials the backgrounds were presented synoptically, i.e. the left eye background picture was displayed to both eyes—and in the other half of the trials the natural pair of background pictures were presented stereoscopically. As this factor was not a significant determinant of other effects, data from the two background conditions were combined. The effect of synoptic vs. stereo-pair backgrounds is discussed further in S5 Notes.

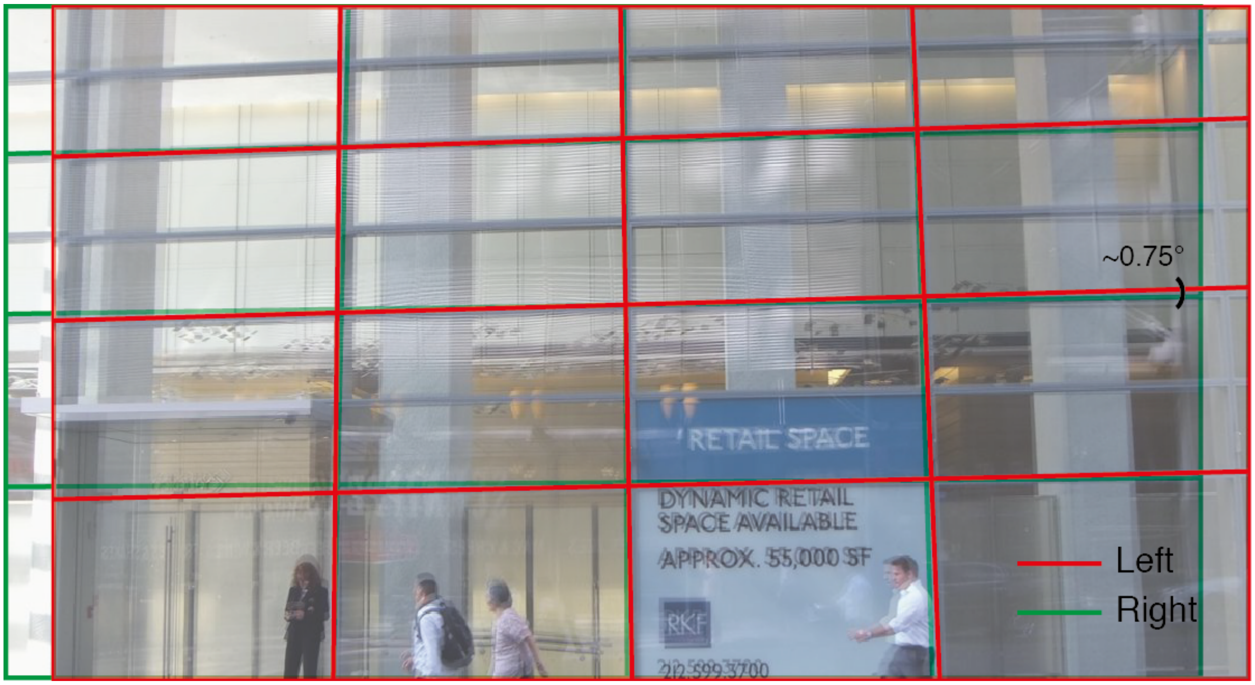

S4 Fig. Super-imposition of left eye and right eye images. The right-eye image is rotated clockwise relative to the left-eye image (excyclodisparity) by about 0.75°.
