# Supplementary material for: Stereoscopic Offset Makes Objects Easier to Recognize
Source: PLoS One. 2015 Jun 16;10(6):e0129101. doi: 10.1371/journal.pone.0129101 (PMC4469586; doi:10.1371/journal.pone.0129101)
Supplement: S3 Notes — (PDF) [file pone.0129101.s006.pdf]

S3 Notes. Effect of the target object.

Performance varied with the target object (S5 Fig.). The sparrow (object 3) and the dirigible (object 6) had recognition rates above 75% on average, while the seagull (object 1) had a recognition rate of only 44%. However, the recognition rate for an object goes up with the bias to select that object as the response; an observer who *always* selected the sparrow would have a recognition rate of 100% for that object and 0% for the other five objects. In Experiment 1 responses were biased and not evenly distributed, with objects 1 to 6 being selected for the response on (1) 10.7 ± 2.4, (2) 16.7 ± 1.3, (3) 19.3 ± 1.6, (4) 20.7 ± 2.9, (5) 16.6 ± 2.3, and (6) 16.0 ± 1.7 percent of trials, respectively (*mean* ± *SD across observers*).

We estimated how discriminable each object was using a measure of sensitivity similar to d-prime. The hit rate for a given object was its recognition rate. Its false alarm rate was the proportion, out of trials on which a different object was presented, that the object was (incorrectly) selected by the observer. Sensitivity was calculated as z(hit rate) – z(false alarm rate), separately for each object for each observer. The mean sensitivity across observers (± *SE*) for the six objects, respectively, were (1) 1.64 ± 0.13, (2) 1.68 ± 0.12, (3) 2.03 ± 0.15, (4) 1.58 ± 0.10, (5) 1.52 ± 0.11, and (6) 2.64 ± 0.16. Thus, the low recognition rate for object 1 was due to bias against this object, not low discriminability; the high recognition rate for object 6 was due mainly to its high discriminability; and the high recognition rate for object 3 was due to both high bias and high discriminability.

To understand which objects were confused with one another, we analyzed the data contingent on the observer having made an error. S6 Fig. represents the confusion matrix for Experiment 1. Gray-values show log (natural) probability ratios (*LPR*), relative to chance.

$$LPR_{i,j} = log\frac{p(o_{j\neq i}|o_i)}{c}$$

where $p(o_{j\neq i}|o_i)$ is the proportion of trials on which object i (o_i) was displayed and object j (o_j) was selected as the answer, with $j \neq i$, and c is the equal-chance level. Here the equal-chance level was 0.2 (probability of 1/5 for choosing each incorrect target). A log-p ratio of 0 indicates that the observers mistakenly reported the object *j* instead of the object *i* on 20% of error trials, as predicted by equal chance. A positive LPR indicates that the object *j* was confounded with (selected in place of) object *i* more often than predicted by equal chance, and a negative value less often than equal chance.

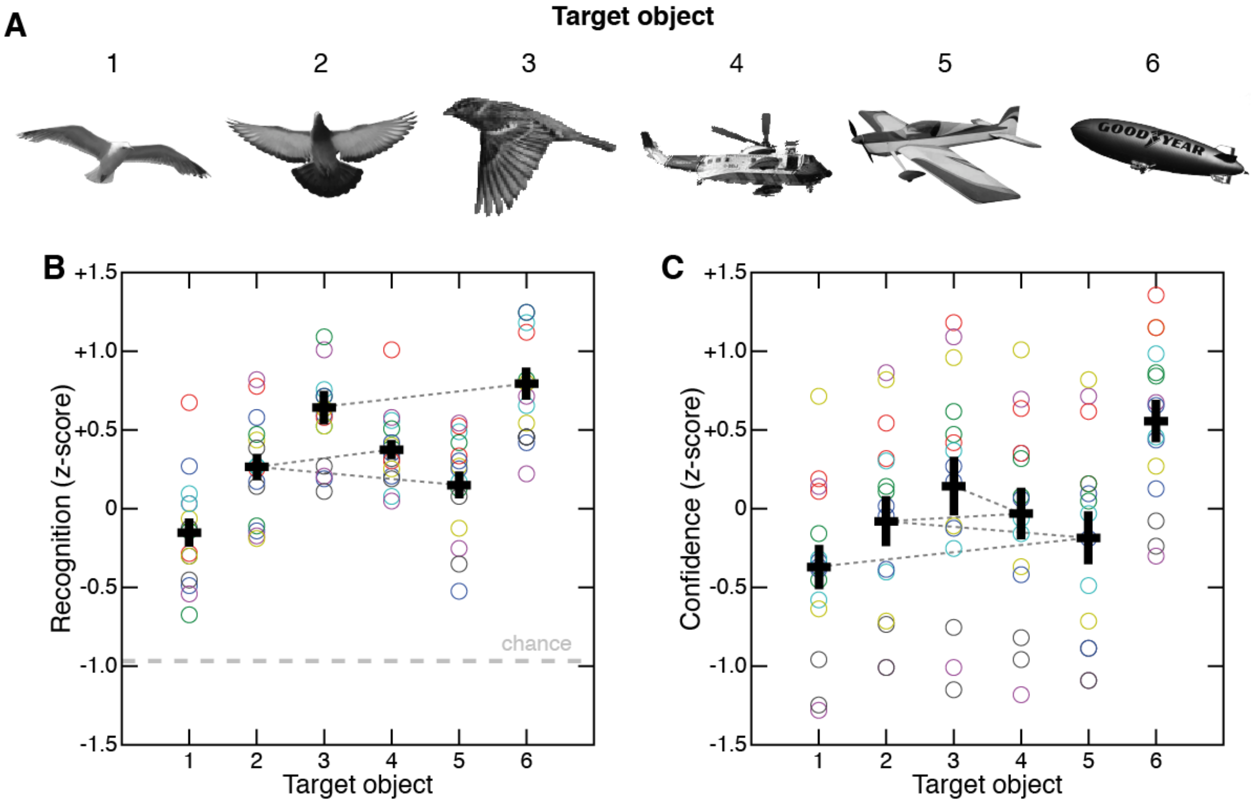

S5 Fig. A: The six target objects used in the study (1-6: seagull, pigeon, sparrow, helicopter, airplane, and dirigible). B: Recognition rates in z-score units as a function of the target object. Colored dots are different observers, horizontal and vertical cross bars show the population mean and its standard error, respectively. Dashed gray lines show the *non*-significant ($p>0.05$) pairwise t-tests with Bonferroni correction for multiple comparisons. C: Same as B for confidence. The recognition rate for an object reflects both its discriminability and the bias to select it when responding.

In S6(A) Fig. the marginal *log p* ratios reflect the objects' false alarm rates, with objects 1 and 6 being less often confounded with targets overall, while object 4 was confounded more often than predicted by chance. S6(B) Fig. plots the LPRs for all dyads of objects. The abscissa is LPR when object *j* was selected for target object *i*, and the ordinate is LPR when object *i* was selected for target object *j*. If the objects were mistakenly reported as predicted by chance alone, all dyads would be at the coordinates (0,0), as is the case for dyads 2-5 (pigeon and plane) and 3-5 (sparrow and plane). All the dyads should lie on the $y=x$ diagonal if objects were equally likely to be mistaken for one another. Object 5 for example was more often mistaken for object 4 than predicted by chance (top-right corner). And reciprocally object 4 was as often mistaken for object 5. But this mutual relationship is true only for the dyads of objects 1-6 (seagull and dirigible), 2-3 (sparrow and pigeon) and 4-5 (helicopter and plane). Most dyads presented strong asymmetries such as the dyad 4-6 (bottom-right corner) where the object 6 (dirigible) was selected for object 4 (helicopter) almost twice as often as predicted by equal chance, but object 4 was selected for object 6 only half as often as

predicted by equal chance. These strong asymmetries in the confusion matrix are likely related to known asymmetries between features in visual search tasks [1].

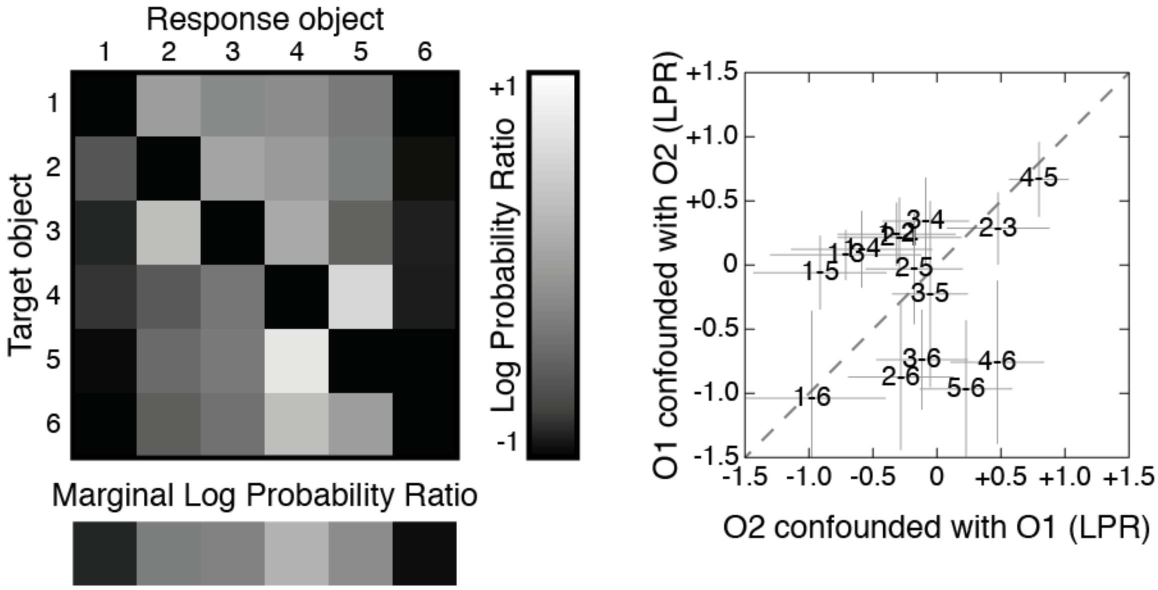

S6 Fig. A: Confusion matrix of the target objects. Values are log p ratios relative to chance when the observer reported an incorrect object. B: Log p ratios for dyads of objects (numbers). The vertical and horizontal lines are standard errors. The y=x diagonal (dashed line) indicates that object 1 is as often mistaken for object 2 as object 2 is mistaken for object 1. If all objects were equally confused, all dyads would be at the 0-0 coordinates (log p ratio of 0, i.e. probability at the equal-chance level of 0.2).

Supplementary references

1. Wolfe JM. Asymmetries in visual search: An introduction. Percept Psychophys. 2001;63: 381–389.
