# Supplementary material for: Stereoscopic Offset Makes Objects Easier to Recognize
Source: PLoS One. 2015 Jun 16;10(6):e0129101. doi: 10.1371/journal.pone.0129101 (PMC4469586; doi:10.1371/journal.pone.0129101)
Supplement: S4 Notes — (PDF) [file pone.0129101.s007.pdf]

S4 Notes. Effect of the target's location.

S7 Fig. represents the 16 possible locations of the target objects on the backgrounds. S8 Fig. shows performance (A) and confidence (B) as a function of the display location.

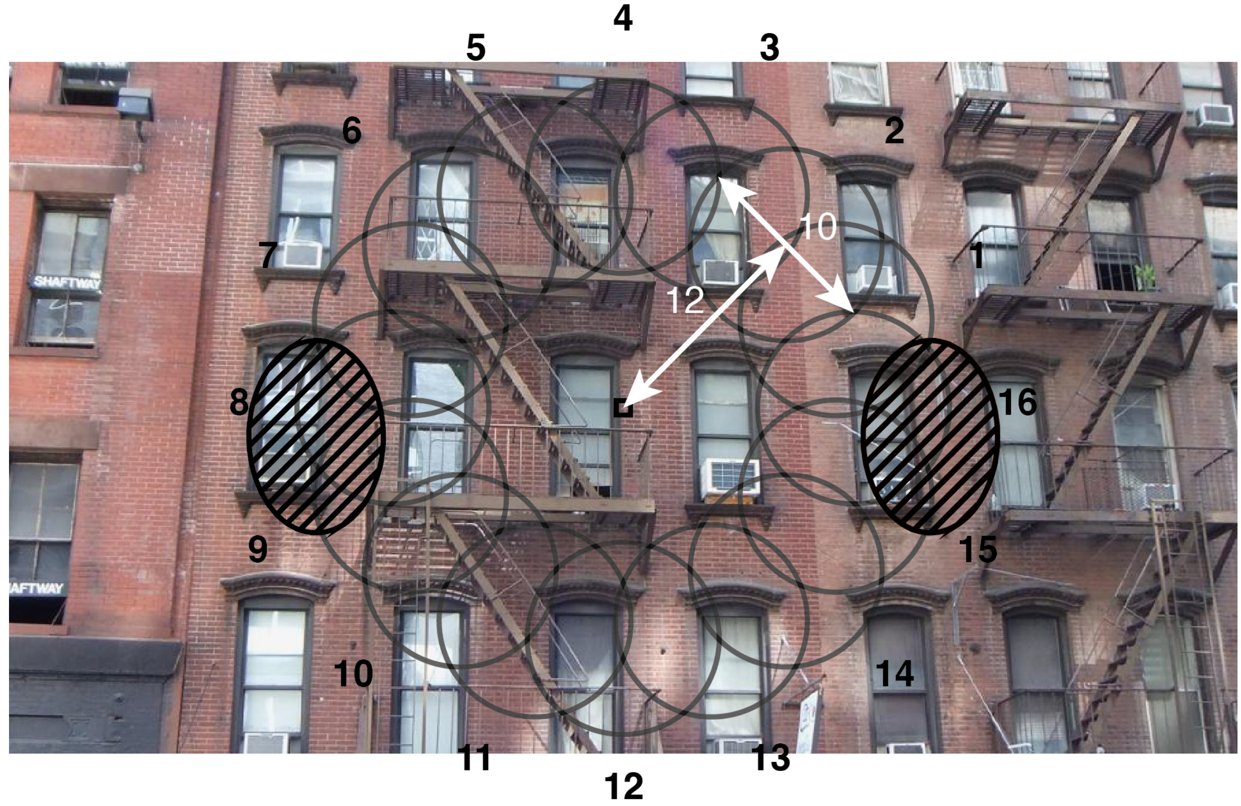

S7 Fig. Possible target locations. The dark semi-transparent circles indicate the 16 possible target locations with their associated location number. The white arrows indicate the stimulus dimensions in degrees of visual angle. The black striped areas represent the approximate location of the right and left eye blind spots.

The target was displayed at one of 16 possible locations. The mean recognition rate for the upper quadrant (locations 2 to 6) and lower quadrant (locations 10 to 14) were respectively 57% and 64%, and were significantly different, $t(14)=3.61$, $p=0.003$. The mean recognition rates for the left and right quadrants (locations 6 to 10 for left, and 14 to 2 for right) were respectively 64% and 65% and were not significantly different, $t(14)=0.38$, $p=0.71$. The mean recognition rate for all upper and lower locations was 61%, which was significantly different from the mean recognition rate of 64% at the left and right locations, $t(14)=6.25$, $p<0.0001$. For confidence judgments the difference between upper and lower quadrants and the difference between left and right were not significantly different, $t(14)=0.06$, $p=0.95$; $t(14)=0.65$, $p=0.52$; respectively. However judgments at the left and right locations were more confident than judgments at upper and lower locations, $t(14)=4.85$, $p=0.0003$.

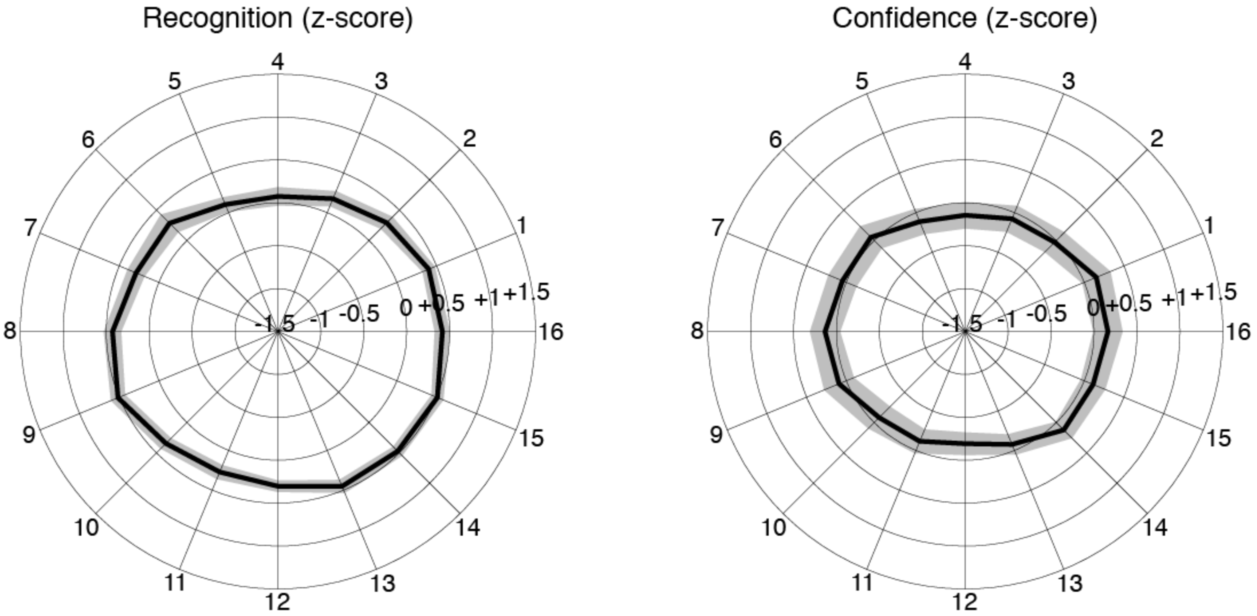

S8 Fig. Recognition and confidence as a function of the target location. The radial lines represent the target location and the distances to the center represent recognition (left) and confidence (right) in z-score from -1.5 (7%) at the center to +1.5 (93%) at the periphery. The thick line is the population average and the gray area the standard error.

A possible explanation for asymmetry in recognition rates across the visual field could be disparity of the target object relative to the horopter. However, the vertical horopter is slanted top far [1–4]; yet performance was better in the lower quadrant, where the targets' crossed disparities were smaller relative to the horopter, than in the upper quadrant where they were larger. The right and left quadrants had similar performance to the lower quadrant rather than being intermediate between the upper and lower. The disparity with peak performance was nearly identical in the 4 separate quadrants: 50, 46 47 and 47 arcmin for the up, down, left and right quadrants respectively. Thus it seems unlikely that the horopter played a role in the observed asymmetries.

Given that attention to spatial location, as recruited by crossed disparity of the target, had a dramatic effect on recognition rates and confidence, it seems more plausible that these asymmetries within the visual field reflect inhomogeneity in the allocation of visual attention.

Supplementary references

1. Von Helmholtz H. Handbuch der physiologischen optik. Leipzig: Leopold Voss; 1867.

2. Nakayama K. Geometric and physiological aspects of depth perception. SPIE. San Diego; 1977. pp. 2–9.

3. Schreiber KM, Hillis JM, Filippini HR, Schor CM, Banks MS. The surface of the empirical horopter. J Vis. 2008;8: 1–20.

4. Cooper EA, Burge J, Banks MS. The vertical horopter is not adaptable, but it may be adaptive. J Vis. 2011;11: 1–19.
