# Supplementary material for: Stereoscopic Offset Makes Objects Easier to Recognize
Source: PLoS One. 2015 Jun 16;10(6):e0129101. doi: 10.1371/journal.pone.0129101 (PMC4469586; doi:10.1371/journal.pone.0129101)
Supplement: S5 Notes — (PDF) [file pone.0129101.s008.pdf]

S5 Notes. Effect of background type.

The camera created artifactual disparities in the stimulus (see S2 Notes). To control for possible effects of these disparities, we used two types of background (i.e. two types of stereo images of building facades). In the first background condition, the “binocular” condition, the 2 monocular pictures taken by the stereo camera were displayed in stereo after adjusting their horizontal positions on the display screen to provide the correct vergence demand for the simulated viewing distance at fixation. In the second background condition, the “synoptic” condition, we displayed the left eye monocular picture to both eyes after horizontal adjustment. In the synoptic condition the backgrounds appeared perfectly flat and orthogonal to the binocular axis. In the binocular condition the building facades contained natural depth modulations, as well as an artifactual relative disparity between the background and the target object, that was introduced by the camera and depended on the target location. Therefore performance might have been different between the 2 background conditions. In particular, the zero-disparity condition actually contained artifactual disparities as large as 9 arcmin between the background and the target when the background was binocular. To determine whether the type of background had an effect, a four-way repeated measures ANOVA was conducted on the z-transformed recognition rates and confidence judgments with duration, disparity, contour and background as factors. For our 15 observers, the background type did not significantly modulate recognition, $F(1,14)=4.25$, $p=0.058$, with the trend towards better performance in the synoptic condition. The interactions with the background type, including its interaction with disparity magnitude, were not significant (S3 Table).

The pattern of significant effects from the ANOVA on z-transformed confidence judgments was identical with one exception (S4 Table): the interaction between disparity, contour and background was statistically significant. The interaction was not obviously meaningful, however: on average across subjects, the confidence rate was 7% lower for the binocular background condition, specifically when disparity was 30 arcmin and the contour was rectangular. There is no theoretical reason to believe that this interaction exists, nor that it would be present for confidence judgments but not recognition rates. Therefore we ignored this effect, as being most likely due to chance.

Because the pattern of results was similar between the two background conditions, and because all the effects described in the main text were present for both background conditions, trials from the two conditions were pooled for the analysis.

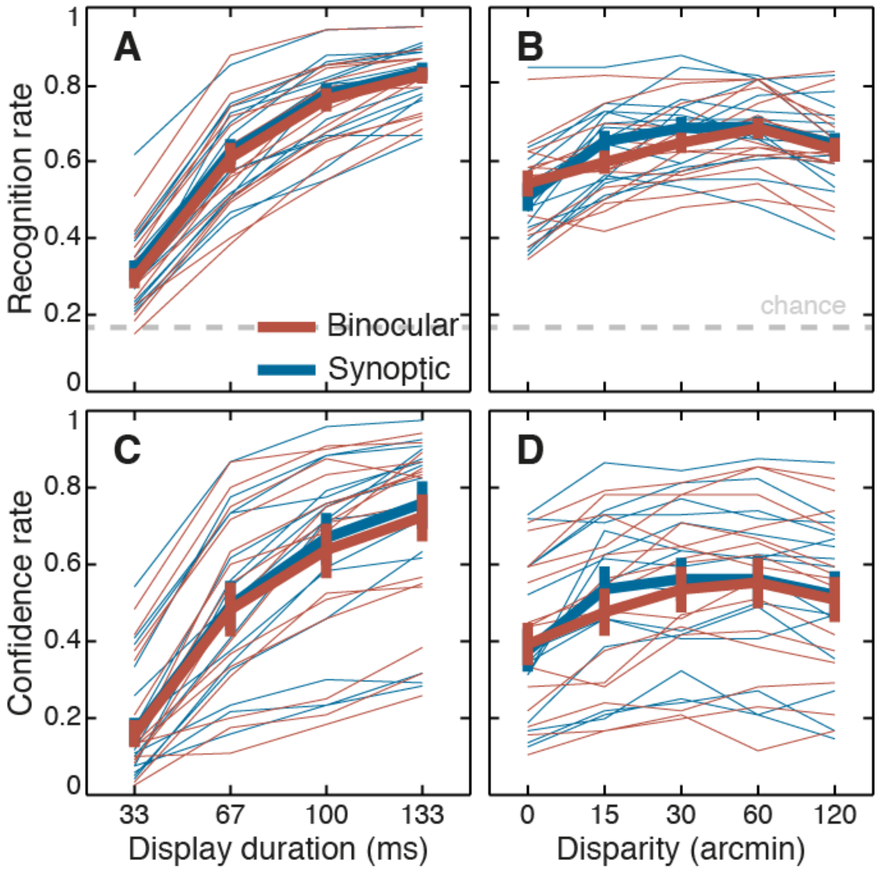

S9 Fig. A: Recognition rate as a function of display duration. B: Recognition rate as a function of disparity. C: Confidence rate as a function of display duration. D: Confidence rate as a function of disparity. Red lines are binocular backgrounds and blue lines are synoptic backgrounds. Thick lines are population average with standard errors, thin lines are individual observers.

S3 Table. Experiment 1, recognition rates.

Effect	F	d.f.	p-value	Sig.
**Duration**	287	3,42	<0.0001	Yes
**Disparity**	6.61	3,42	0.0009	Yes
**Contour**	18.3	1,14	0.0008	Yes
**Background**	4.25	1,14	0.058	No
**Duration & Disparity**	0.159	9,126	1.0	No
**Duration & Contour**	3.41	3,42	0.026	Yes
**Duration & Background**	0.372	3,42	0.77	No
**Disparity & Contour**	0.577	3,42	0.63	No
**Disparity & Background**	0.349	3,42	0.79	No
**Contour & Background**	0.818	1,14	0.38	No
**Duration & Disparity & Contour**	1.03	9,126	0.42	No
**Duration & Disparity & Background**	0.822	9,126	0.60	No

**Duration & Contour & Background**	0.531	3,42	0.66	No
**Disparity & Contour & Background**	1.59	3,42	0.21	No
**Duration & Disparity & Contour & Background**	1.54	9,126	0.14	No

Results of the ANOVA on recognition rates. The ampersand indicates an interaction. The column "F" reports F-values, the column "d.f." reports degrees of freedom and the column "p-value" reports the p-values.

S4 Table. Experiment 1, confidence judgments.

Effect	F	d.f.	p-value	Sig.
**Duration**	111	3,42	<0.0001	Yes
**Disparity**	8.20	3,42	0.0002	Yes
**Contour**	8.43	1,14	0.012	Yes
**Background**	2.73	1,14	0.12	No
**Duration & Disparity**	0.782	9,126	0.63	No
**Duration & Contour**	2.87	3,42	0.048	Yes
**Duration & Background**	0.89	3,42	0.45	No
**Disparity & Contour**	1.58	3,42	0.21	No
**Disparity & Background**	0.970	3,42	0.42	No
**Contour & Background**	0.850	1,14	0.37	No
**Duration & Disparity & Contour**	0.996	9,126	0.45	No
**Duration & Disparity & Background**	0.886	9,126	0.54	No
**Duration & Contour & Background**	0.309	3,42	0.82	No
**Disparity & Contour & Background**	6.67	3,42	0.0009	Yes
**Duration & Disparity & Contour & Background**	1.29	9,126	0.25	No

Results of the ANOVA on confidence judgments. The ampersand indicates an interaction. The column "F" reports F-values, the column "d.f." reports degrees of freedom and the column "p-value" reports the p-values.
