# Supplementary material for: Stereoscopic Offset Makes Objects Easier to Recognize
Source: PLoS One. 2015 Jun 16;10(6):e0129101. doi: 10.1371/journal.pone.0129101 (PMC4469586; doi:10.1371/journal.pone.0129101)
Supplement: S6 Notes — (PDF) [file pone.0129101.s009.pdf]

## S6 Notes. Effect of disparity sign.

By accident one observer ran Experiment 1 with reversed phases on the shutter glasses (i.e. the left eye became occluded when the left image was displayed and became clear when the right image was displayed, and vice versa for the right eye). Consequently all disparities were inverted in sign, similar to wearing a pseudoscope. The backgrounds had a crossed disparity relative to the display screen and the target objects had an uncrossed disparity relative to the backgrounds. Results for that observer (see S10 Fig.) showed a reversed pattern of the effect of disparity compared to the other observers, with performance decreasing rather than increasing when disparity was added to the target (compare to Fig. 6 and S2 Fig. in S1 Notes). Performance still increased with display duration. These data suggest that it is not disparity per se, but rather disparity-specified nearness that attracts attention to the location of the target within the visual field. However, in this stimulus, occlusion cues specified a near object so they conflicted with disparity, and this may have limited the efficacy with which disparity attracted attention or defined a contour. It is known, for example, that the response of cortical neurons that are tuned to contour are modulated by boundary ownership: a given contour will elicit greater neuronal response when it is a "real" bounding contour belonging to a foreground (occluding) shape, than when it is an "accidental" contour of an occluded shape [1–3]. Activity in these neurons would have been disrupted by the reverse-disparity artifact.

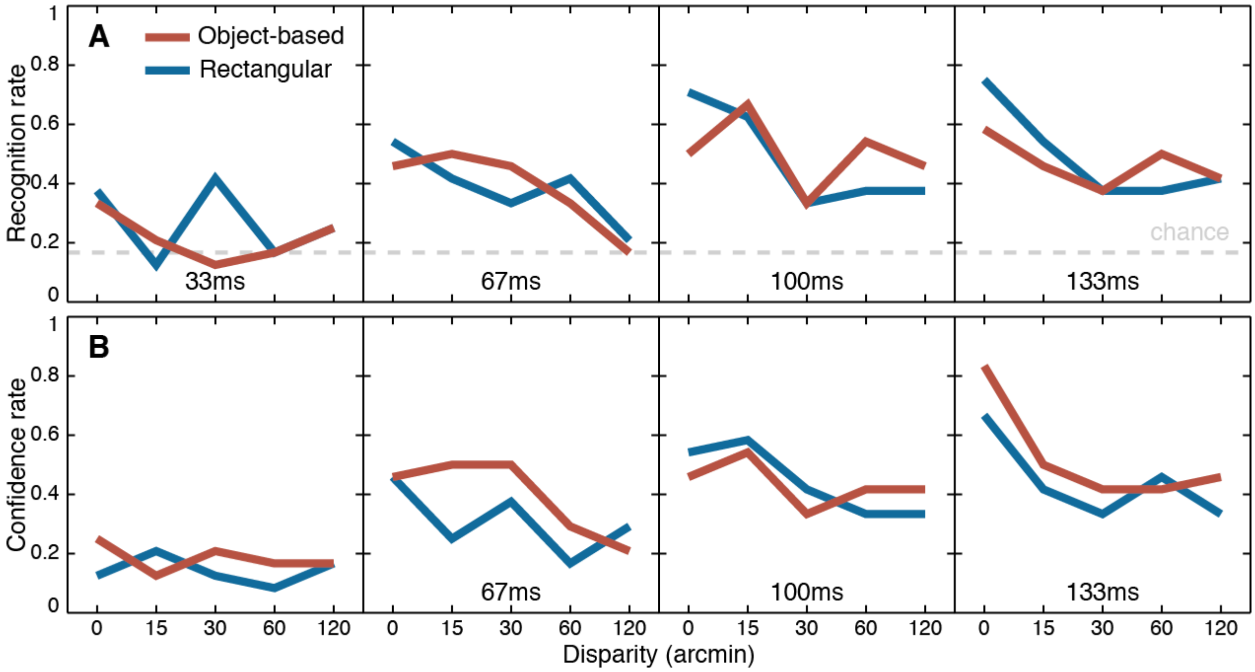


**S10 Fig.** Effect of pseudoscopic vision. A: Lattice plot of the recognition rate as a function of disparity (in abscise) and display duration (from left to right: 33, 67, 100 and 133ms) for the object-based stereoscopic contour condition (red lines) and rectangular stereoscopic contour condition (blue lines). B: Same for confidence rates.

**Supplementary references**


1. Bushnell BN, Harding PJ, Kosai Y, Pasupathy A. Partial Occlusion Modulates Contour-Based Shape Encoding in Primate Area V4. J Neurosci. 2011;31: 4012–4024.

2. Qiu FT, von der Heydt R. Figure and Ground in the Visual Cortex: V2 Combines Stereoscopic Cues with Gestalt Rules. Neuron. 2005;47: 155–166.

3. Von der Heydt R, Zhou H, Friedman HS. Representation of stereoscopic edges in monkey visual cortex. Vision Res. 2000;40: 1955–1967.
